# *De novo* Genome Assembly and Annotation of 12 Fungi Associated with Fruit Tree Decline Syndrome in ON, Canada

**DOI:** 10.1038/s41597-025-05192-5

**Published:** 2025-07-01

**Authors:** Muhammad Sulman, Evgeny Ilyukhin, Oscar Villanueva, Hai D. T. Nguyen, Shawkat Ali, Walid Ellouze

**Affiliations:** 1 Agriculture and Agri-Food Canada, Vineland Station, Ontario L0R 2E0 Canada; 2 Agriculture and Agri-Food Canada, 960 Carling Avenue, Ottawa, Ontario K1A 0C6 Canada; 3 Agriculture and Agri-Food Canada, Kentville, Nova Scotia B4N 1J5 Canada

**Keywords:** Fungal genomics, Comparative genomics

## Abstract

Apple and stone fruit trees are vital components of Ontario’s agricultural landscape. However, since 2016, these trees have been facing alarming mortality rates, exhibiting symptoms collectively referred to as Fruit Tree Decline (FTD) and Rapid Apple Decline (RAD). Despite its widespread occurrence, the exact cause of FTD and RAD remains elusive, with various pathogenic fungi and viruses implicated, along with abiotic stressors such as drought, winter injury and nutrient deficiency. In this study, we sequenced, assembled and annotated the genomes of 12 fungi associated with FTD and RAD syndromes in Ontario, Canada. We present the first and only publicly available assemblies for three ascomycete species including *Diplodia intermedia*, *Diatrype stigma*, and *Nothophoma quercina*. Additionally, we present high-quality reference genome sequences for *Diplodia seriata*, *Didymella pomorum* and *Neofusicoccum ribis*. These genomic resources are valuable for understanding the molecular mechanisms behind FTD and RAD, and for developing strategies for disease prevention and management in fruit trees.

## Background & Summary

Apple and stone fruit trees are integral to Ontario’s agriculture economy. The primary areas for apple production in Ontario are located along the shores of Lake Ontario, Lake Erie, Lake Huron and the Georgian Bay. Recent years have seen a concerning downturn, with apple cultivation decreasing to 6,414 hectares in 2023, a 28% drop from the 8,903 hectares recorded in 2002^1^. Similarly, the Niagara region, responsible for over 90% of Ontario’s stone fruit production, has seen a decrease in cultivation area, down 37% from 5,080 hectares in 2007 to 3,192 hectares in 2023^1^.

Since 2016, both apple and stone fruit trees have faced alarming levels of mortality in Ontario, with reports of up to 42% mortality in apples and up to 72% in stone fruits, particularly affecting trees aged between 2 to 10 years^2–8^. Symptoms of decline vary, from leaf discoloration and canker development in apples to progressive dieback in stone fruits. These symptoms collectively define Fruit Tree Decline (FTD), with Rapid Apple Decline (RAD) specifically identified in apples^9^. The decline observed in apple and stone fruit trees extends beyond Ontario, echoing in other regions across North America^10^. While the exact cause of FTD remains elusive, the emergence of new pathogenic fungi^2–8,11^ and viruses^12–14^, as well as the re-emergence of known pathogens^15–17^, compounded by unexpected abiotic stressors such as drought stress, heat waves, floods, winter injury, and nutrient deficiency, are thought to play a significant role in its development^9,18,19^.

Fungal pathogenicity in a plant depends on multiple factors, including the specific traits of the pathogen, the plant’s defenses, and the conditions within their shared environment^20^. While weak pathogens may individually induce negligible FTD symptoms, the severity of the disease can escalate under the influence of other biotic or abiotic stresses. Understanding these interactions and the underlying genetic mechanisms is key for effective disease management.

Sequencing fungal genomes lays the foundation for exploring their ecological niche, evolutionary patterns, and disease-causing capabilities^21^. By deciphering the genetic makeup of these fungi, we aim to improve disease diagnosis, elucidate pathogenic mechanisms, and support the development of targeted strategies for disease prevention and management^22^. In the present study, we sequenced, *de novo* assembled and annotated the genomes of 12 fungi associated with FTD and RAD syndromes in Ontario, Canada, spanning a spectrum from pathogens to weak pathogens and non-pathogens/endophytes. By identifying genetic differences between pathogens and non-pathogens, we can gain a stronger understanding into the genetic determinants driving pathogenic evolution^23^. This knowledge is required for developing predictive models for fungal pathogen emergence and designing effective control measures.

This study provides the first assembled and annotated genomes for the Ascomycetes species *Diplodia intermedia*, *Diatrype stigma*, and *Nothophoma quercina*. These represent the only genome assemblies currently available for the *Diatrype* and *Nothophoma* genera. We also present high-quality and well-annotated reference genome for *Diplodia seriata*, *Didymella pomorum* and *Neofusicoccum ribis*. These new genomic resources significantly enhance our ability to study fungal pathogenicity in agricultural systems and inform the development of targeted control measures against pathogens. Moreover, the availability of these assemblies enables downstream functional analyses, including the identification of effector proteins, which are key virulence factors used by pathogens to facilitate host colonization^24^. Since effector characterization can inform resistance breeding and integrated disease management strategies, this work supports future discovery efforts and comparative genomic studies aimed at improving the resilience and sustainability of fruit production systems.

## Methods

### Sample collection, fungal isolation and identification

Between 2018 and 2021, wood samples were collected from 25 apple, 30 apricot, six peach, and six nectarine trees showing extensive symptoms of tree fruit decline across 15 commercial orchards in Ontario. Tree mortality rates reached up to 42% in apples and up to 72% in stone fruits, with the highest impact observed in trees between 2 and 10 years of age across the sampled orchards. Small sections of diseased wood (1 cm long) underwent surface sterilization with 70% ethanol for 30 seconds, followed by treatment with 1% NaClO for 20 minutes. After rinsing thrice in sterile water, the wood samples were placed on 2% potato dextrose agar (PDA, Difco™, Franklin Lakes, NJ, USA) with kanamycin (50 mg/L) and incubated at 22 °C for 5 days in darkness. Fungal colonies consistently isolated underwent hyphal-tip transfer to individual PDA plates, then incubated at 22 °C for 7 days in darkness. Purified isolates were morphologically classified into morphotypes before molecular identification.

Genomic DNA was extracted from the mycelium grown on PDA of 7-day-old cultures of one representative isolate from each morphotype group using the Plant/Fungi DNA Isolation Kit (Norgen Biotech, ON, Canada, cat. no. 26200), following the manufacturer’s instructions with slight modifications. Specifically, fungal tissue was vortexed for 15 minutes with 1 mm glass beads in 500 μL of lysis buffer supplemented with 1 μL of RNase A, followed by incubation at 65 °C for 10 minutes. Subsequently, 100 μL of Binding Buffer I was added, the mixture was thoroughly mixed, and incubated on ice for 5 minutes before centrifugation at 10,000 rpm to separate the lysate from the beads and biomass. Resin drying during the column wash was achieved by spinning for 10 minutes at 14,000 rpm. Finally, DNA was eluted at 10,000 rpm for 2 minutes.

Polymerase chain reaction (PCR) was performed in a VWR PCR Thermal Cycler XT^96^ Gradient (VWR, USA) under the following conditions: 98 °C for 30 sec; 35 × (98 °C, 10 sec; 60 °C, 30 sec; 72 °C, 30 sec); 72 °C, 2 min. The internal transcribed spacer (ITS), translation elongation factor 1-α (EF1-α) and β-tubulin (TUB2) gene regions were each amplified in separate PCR reactions using the primers ITS1/ITS4^25^, EF1-728 F/EF1-986R^26^ and Bt2a/Bt2b^27^, respectively. Each 30 μL reaction contained 9 μL of nuclease-free Water, 15 μL of Q5 Hot Start High-Fidelity 2X Master Mix (New England Biolabs, ON, Canada, cat. no. M0494S), 3 μL of extracted genomic DNA and 1.5 μL (10 μM stock) of the appropriate fungal-specific primers. Reactions were performed with negative controls containing no DNA.

The quality of the PCR products was examined using electrophoresis in 1% agarose gel. Sanger sequencing was carried out at Genome Quebec’s Sequencing Facility (Montreal, Canada). Sequences were analyzed with the Basic Local Alignment Search Tool (BLAST)^28^ against the nucleotide database for an early approximation of species classification.

### DNA isolation and genome sequencing

Genomic DNA was extracted from fungal mycelium obtained from 12 pure cultures of fungi identified as previously described. These fungi include strains such as *Botryosphaeria dothidea*, *Cytospora paraplurivora*, *Diaporthe eres*, *Diatrype stigma*, *Didymella pomorum*, *Diplodia intermedia*, *Diplodia seriata*, *Neofusicoccum ribis*, *Nothophoma quercina*, and *Paraconiothyrium brasiliense*, all isolated from symptomatic apple, apricot, and nectarine trees (Table 1). Eleven of the 12 strains were deposited at the Canadian Collection of Fungal Cultures (DAOMC), each assigned a unique identification number as detailed in Table 1. The mycelium was cultured in potato dextrose broth (PDB, Difco™, Franklin Lakes, NJ, USA) for 5 days at room temperature on an orbital shaker (3 g), followed by filtration through sterilized Whatman^TM^ Qualitative filter paper grade 1 (Cytiva, Marlborough, MA, USA), freeze-drying for 48 hours using a Benchtop Freeze Dry System (Labconco FreeZone^®^ 4.5 Liter, Kansas City, MO, USA), and bead beating using 1 mm glass beads until a powdered form was obtained. Subsequently, the modified DNA extraction protocol, as previously described (Norgen Biotech, Thorold, ON, Canada), was employed.

**Table 1:** Genome assembly and annotation statistics.

Organism (DAOMC#)	Accession	Length (bp)	Sequencing Method	Coverage	Contigs	N50 (bp)	L50	GC percent	Protein-coding Genes	Non-coding Genes
*Botryosphaeria dothidea* M68-17 (DAOMC252246)	GCA_021436945.2	45,122,761	Illumina NovaSeq	136.6x	657	286,094	47	55	12,423	130
*Cytospora paraplurivora* FDS-564 (DAOMC252466)	GCA_021272945.2	39,445,691	PacBio Sequel II	170.0x	187	658,741	18	51	9,349	192
*Diaporthe eres* M169 (DAOMC252249)	GCA_022570805.2	61,247,700	Illumina NovaSeq	112.3x	1,202	351,496	48	49	14,067	157
*Diaporthe eres* M63-4 (DAOMC252250)	GCA_022570775.2	59,241,446	PacBio Sequel II	243.2x	162	644,873	24	50	14,171	167
*Diatrype stigma* M11/M66-122 (DAOMC252251)	GCA_022225965.2	49,153,611	Illumina NovaSeq	145.9x	420	241,545	64	49	11,292	163
*Didymella pomorum* M27-16 (DAOMC252252)	GCA_022225945.2	35,360,947	Illumina NovaSeq	281.9x	423	403,404	29	54	10,116	158
*Diplodia intermedia* M45-28 (DAOMC252253)	GCA_021495925.2	38,098,648	Illumina NovaSeq	235.3x	253	289,184	39	56	11,222	149
*Diplodia seriata* FDS-637	GCA_021436955.3	37,536,239	PacBio Sequel II and Illumina NovaSeq	427.4x	21	3,256,270	5	57	10,553	154
*Diplodia seriata* M28-159 (DAOMC252254)	GCA_021436965.2	37,023,545	Illumina NovaSeq	184.4x	186	481,311	26	57	10,624	153
*Neofusicoccum ribis* M1-105 (DAOMC252247)	GCA_021436925.2	43,226,643	Illumina NovaSeq	179.0x	659	155,857	74	57	12,143	127
*Nothophoma quercina* M97-236 (DAOMC252260)	GCA_021642125.2	34,358,614	Illumina NovaSeq	227.5x	181	833,360	16	52	9,970	146
*Paraconiothyrium brasiliense* M42-189 (DAOMC252261)	GCA_022225985.2	39,345,625	Illumina NovaSeq	199.7x	108	1,183,519	11	52	12,000	122

Sequencing strategies were designed to species abundance and data requirements. *Diplodia seriata* FDS-637, one of the most abundant species in the study, was sequenced using both PacBio and Illumina platforms to generate high-quality long-read data for comprehensive genome assembly. Two additional abundant species, *Cytospora paraplurivora* FDS-564 and *Diaporthe eres* M63-4 were sequenced using PacBio only, while the remaining nine isolates were sequenced using Illumina, which provided sufficient resolution.

Genomic DNA (gDNA) libraries for *Cytospora paraplurivora* FDS-564, *Diaporthe eres* M63-4, and *Diplodia seriata* FDS-637 were constructed and sequenced at the SickKids sequencing facility (Toronto, ON, Canada). Combinations of barcoded primers and SMRTbell adapters were used to prepare the samples, which were afterward pooled. Long reads for genome assembly were generated from a library prepared with 5 µg of unsheared gDNA using the Express Template Prep Kit (v2.0) (Pacific Biosciences, Menlo Park, CA). A post-library size-selection step targeting fragments > 14 kb was performed using the Agilent 4200 TapeStation System (Agilent, Waldbronn, Germany). The final size-selected library was sequenced on a PacBio Sequel^®^ II sequencer using a single 8 M SMRT Cell in continuous long-read (CLR) mode with a 15-hour movie acquisition time. Raw reads were processed using PacBio’s *P-filter* to remove low-quality reads and adapter sequences.

Illumina gDNA libraries for *Diplodia seriata* FDS-637 and the remaining nine fungal isolates were prepared and sequenced at the Centre d’Expertise et de Services, Génome Québec, McGill University (Montréal, QC, Canada). Genomic DNA was quantified using the Quant-iT™ PicoGreen® dsDNA Assay Kit (Life Technologies™, Grand Island, NY, USA), and its integrity was assessed on a TapeStation 2200 (Agilent Technologies, Inc. Santa Clara, CA, USA). Libraries were prepared using the NEBNext Ultra II DNA Library Prep Kit for Illumina (New England Biolabs, Whitby, ON, Canada) according to the manufacturer’s instructions. Adapters and PCR primers were purchased from Integrated DNA Technologies (IDT, Coralville, IA, USA). Size selection to obtain the desired insert size was performed using sparQ beads (Quantabio, Beverly, MA, USA). Library quantification was carried out using the KAPA Library Quantification Universal Complete Kit (Kapa Biosystems; Wilmington, MA, USA), and average fragment size was determined using a LabChip GX II instrument (PerkinElmer, Waltham, MA, USA). Libraries were normalized, pooled, denatured with 0.02 N NaOH, and neutralized using HT1 buffer. The pooled libraries were loaded at 225 pM on an Illumina NovaSeq 6000 S4 flow cell using the Xp protocol, following the manufacturer’s guidelines. Sequencing was conducted in paired-end mode (2 × 150 bp cycles). A 1% PhiX control library was spiked into the run. Base calling was performed using Illumina Real-Time Analysis (RTA) software (v3), and demultiplexing and FASTQ file generation were done using bcl2fastq2 Conversion Software (v2.20) (Illumina, San Diego, CA, USA).

### Genome *de novo* assembly and annotation

Illumina NovaSeq reads (shown in Table 1) were trimmed with Trimmomatic (v0.38.1)^29^. The ILLUMINACLIP setting was used to remove adapter sequences, and a sliding window approach (4 bp window with an average quality threshold of Q = 20) was applied to trim low quality base calls. The quality of the reads were analyzed before and after Trimmomatic by FastQC (v0.72). Trimmed reads were assembled and gaps were filled using SPAdes (v3.12.0)^30^ with the K-mer values 21, 33, 45, 69, 81, 93, 105, and 117.

PacBio reads for *Cytospora paraplurivora* FDS-564, *Diaporthe eres* M63-4 and *Diplodia seriata* FDS-637 were trimmed and assembled using Canu (v2.1.1)^29^ with default settings, as described by Ilyukhin *et al*.^8^. For *Diplodia seriata* FDS-637, Illumina paired-end reads were aligned to the Canu-assembled PacBio genome using Burrow Wheeler Alignment (BWA (v0.7.17))^31^ with default settings to generate a SAM file. This file was then converted to a sorted BAM file using SAMtools (v1.14)^32^, which served as input for Pilon (v1.23)^33^ to correct base-level errors and enhance the overall accuracy of the Canu assembly. Quast (v5.0.2)^34^ was used to determine assembly statistics and genome coverage (Table 1).

The Funannotate (v1.8.18)^35^ pipeline was used for genome annotation following the recommended instructions (https://funannotate.readthedocs.io) for gene prediction. All assemblies were cleaned and masked using Funannotate default settings, which utilize Tantan (v40)^36^ (https://gitlab.com/mcfrith/tantan) for repeat masking. Subsequently, Benchmarking Universal Copy Orthologs (BUSCO (v2.0.0))^37^ was run on all genomes using the dikarya_odb9 dataset and its results were used to train GlimmerHMM (v3.0.4)^38^, SNAP (2006-07-28)^39^, and Augustus v3.3.3^40^ for *ab initio* gene prediction. Gene predictions were generated using the aforementioned trained gene models and GeneMark-ES (v4.59)^41^. Weights were assigned to each prediction, based on the source and the strength of each prediction, and passed into EVidenceModeler^42^, which was used to generate a consensus gene model. Finally, tRNAscan-SE^43^ was used to generate tRNA predictions.

Functional Annotation was performed using the funannotate wrapper through which the results of numerous analyses were combined. Secondary metabolic gene clusters were predicted using antiSMASH (v6.0)^44^. The number of each category of biosynthetic gene clusters, found by antiSMASH are listed in Table 2. Interproscan (v5)^45^, eggnog-mapper (v2)^46^ (utilizing eggNOG (v5.0)^47^), HMMer3 searches against Pfam (v32.0)^48^, dbCAN (v8.0)^49^; and diamond searches against MEROPS (v12.0)^50^, uniprot and CAZYmes^51^ databases were used to annotate protein functional domains. Phobius (v1.01)^52^ was used to predict protein secretion and transmembrane domains, while signal peptides were predicted using SignalP (v6.0)^53^. The number of annotations added by these steps is summarized in Table 3.

**Table 2:** Counts of different biosynthetic gene clusters found from antiSMASH, in each genome.

Organism	Terpene	T1PKS	NRPS-like	NRPS	Betalactone	Indole	Fungal-RiPP	T3PKS	Siderophore	Other
*Botryosphaeria dothidea* M68-17	12	28	18	19	2	0	1	0	0	0
*Cytospora paraplurivora* FDS-564	11	27	14	11	0	4	1	2	0	1
*Diaporthe eres* M169	14	58	19	20	0	7	1	2	1	1
*Diaporthe eres* M63-4	12	51	17	20	0	5	1	2	1	1
*Diatrype stigma* M11/M66-122	10	21	12	7	0	3	0	1	1	0
*Didymella pomorum* M27-16	4	5	7	2	1	1	0	0	0	0
*Diplodia intermedia* M45-28	7	13	10	4	1	0	0	0	0	0
*Diplodia seriata* FDS-637	7	7	8	3	1	0	0	0	0	0
*Diplodia seriata* M28-159	8	8	9	3	1	0	0	0	0	0
*Neofusicoccum ribis* M1-105	12	22	13	19	1	0	0	0	0	0
*Nothophoma quercina* M97-236	4	3	5	4	0	0	0	0	0	0
*Paraconiothyrium brasiliense* M42-189	10	15	7	8	0	2	0	0	0	0

**Table 3:** Protein counts with functional annotations from each of the listed databases.

Organism	GO Ontology	InterProScan	EggNOG	Pfam	CAZyme	Merops	Secretion
*Botryosphaeria dothidea* M68-17	6,917	9,496	11,435	8,494	581	416	1,092
*Cytospora paraplurivora* FDS-564	5,480	7,445	8,811	6,659	411	334	601
*Diaporthe eres* M169	7,747	10,805	12,997	9,564	814	525	1,313
*Diaporthe eres* M63-4	7,715	10,780	13,090	9,329	790	514	1,266
*Diatrype stigma* M11/M66-122	6,237	8,612	10,475	7,491	562	379	935
*Didymella pomorum* M27-16	5,831	7,899	9,595	7,209	550	363	839
*Diplodia intermedia* M45-28	6,121	8,407	10,169	7,455	494	342	954
*Diplodia seriata* FDS-637	5,879	8,028	9,702	7,003	478	335	814
*Diplodia seriata* M28-159	5,901	8,068	9,769	7,091	486	345	825
*Neofusicoccum ribis* M1-105	6,876	9,455	11,334	8,469	589	408	1,025
*Nothophoma quercina* M97-236	5,740	7,755	9,499	6,909	513	357	792
*Paraconiothyrium brasiliense* M42-189	6,453	8,966	10,970	7,777	630	425	966

Secretion refers to the number of proteins predicted to have signal peptides by SignalP.

**Fig. 1:**
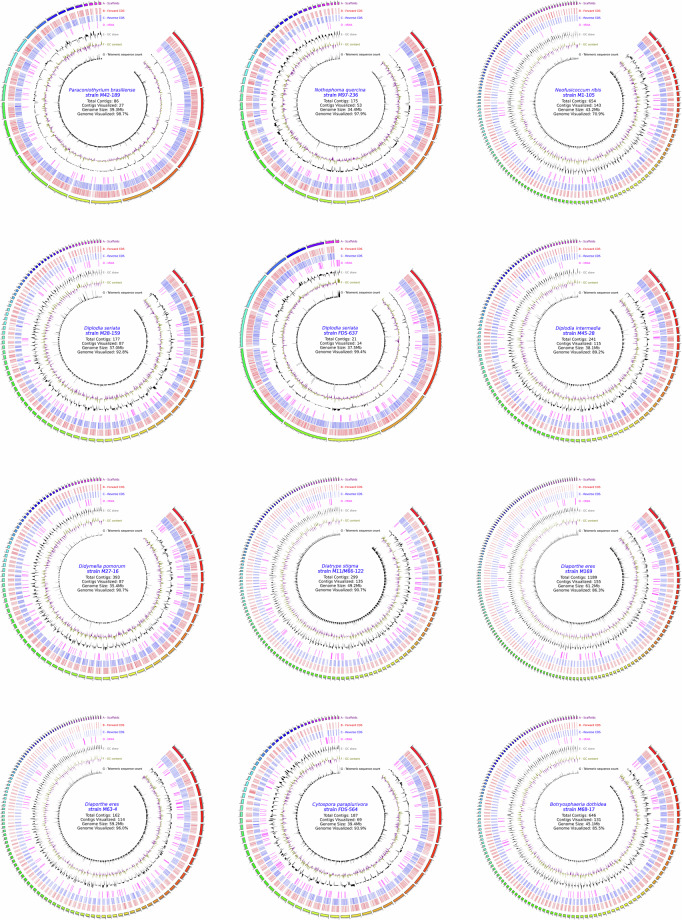
Circos plots for all fungal assemblies in this study, each with 7 rings lettered A to G. Only scaffolds greater than 100 kbp are shown. **Ring A** illustrates scaffolds in the assembly, with smaller ticks are placed at intervals of 100 kbp, and larger ticks are placed at intervals of 1Mbp. **Ring B** highlights the coding sequences (CDSs) on the forward strand, while **Ring C** does the same for the reverse strand and **Ring D** depicts the tRNAs. **Ring E** presents the direction and magnitude of GC skew, with black fill signifying an increase in GC skew relative to the average of the entire genome, while gray represents a decrease. Similarly, **Ring F** presents the GC content, with olive fill representing an increase in GC content over the average of the assembly, while magenta represents a decrease. **Ring G** plots the counts of telomeric sequence.

**Fig. 2:**
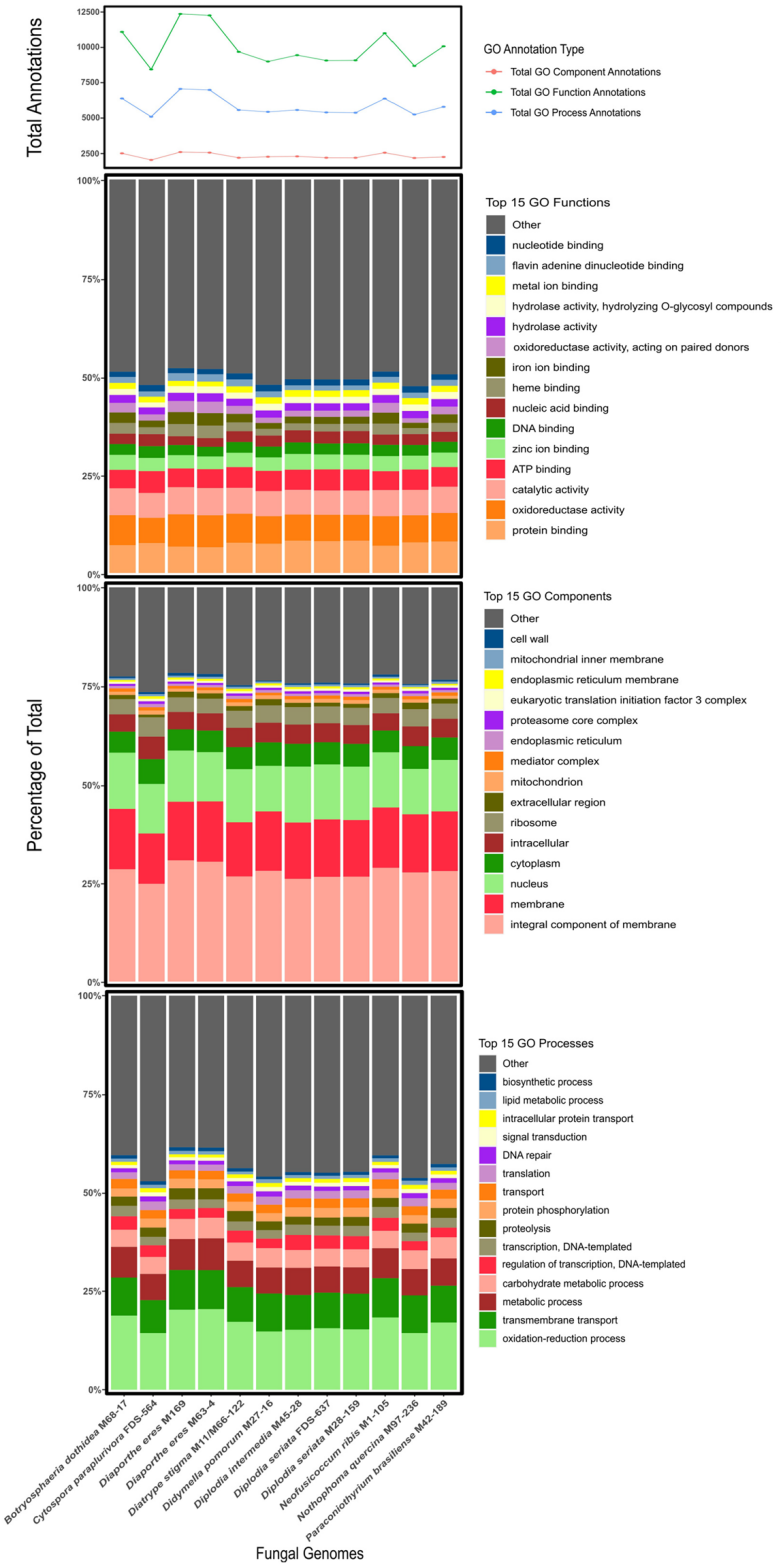
A series of plots representing the number of GO ontology terms in each annotation. In order of top to bottom, the first plot shows a line graph for the number of instances of the three different categories of GO terms: GO process, referring to the biological processes in which a particular gene may play a part; GO function, the activity the product of the gene may possess; and GO component, the location where the gene product is active. The next three plots (in order: GO function, GO component, and GO process) represent the relative percentages of each of the top 15 most common GO terms from their respective category, while terms not in the top 15 are summarized in the “other” group.

**Fig. 3:**
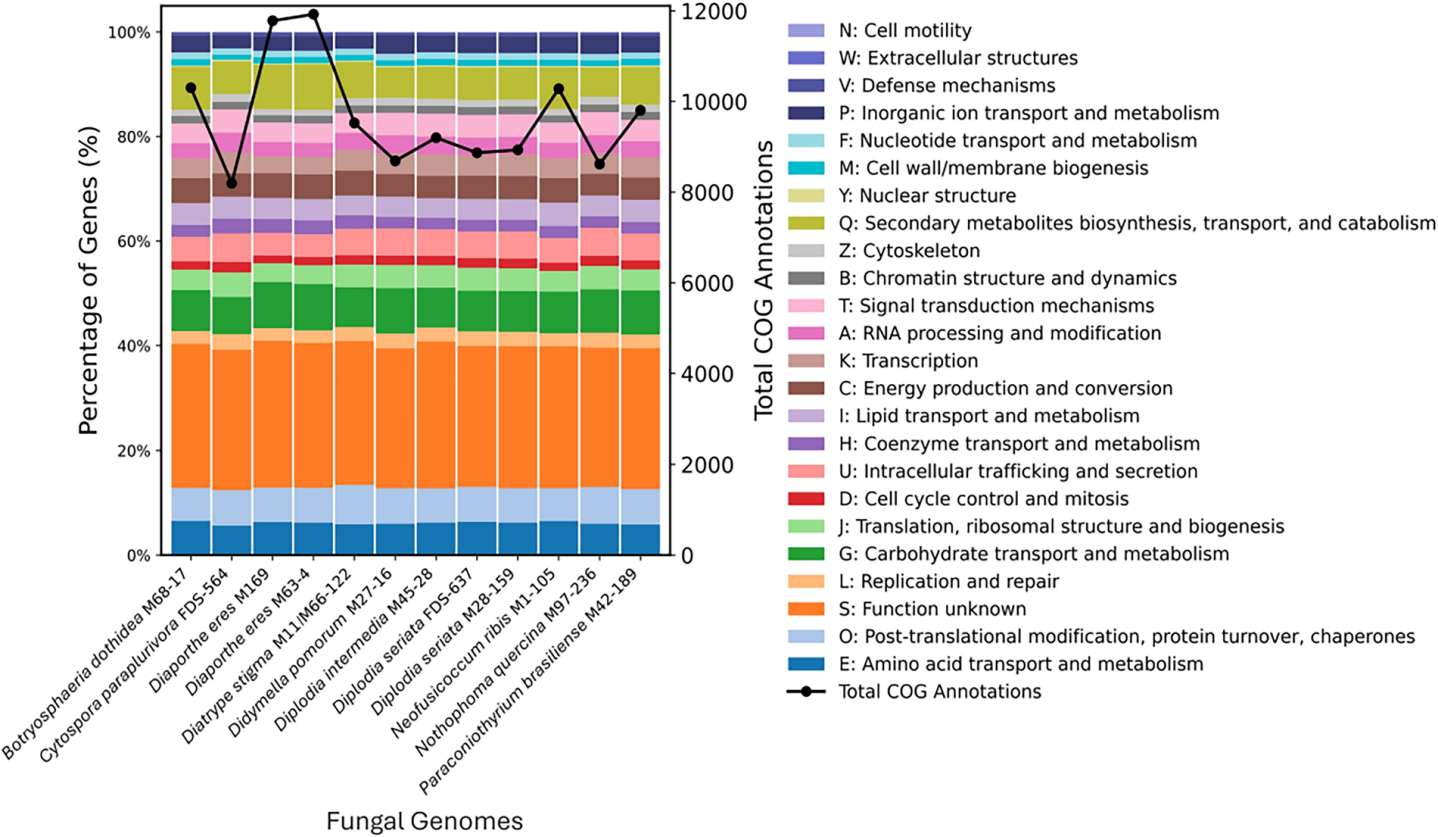
Plot of COGs in each annotation. Each bar represents the percent number of each of the COGS (the corresponding y-axis is on the left). The line graph shows the total number of COG annotations in each assembly (with the corresponding y-axis on the right).

Genomes were visualized using Circos diagrams to assess quality (Fig. 1). GC skew and GC content were calculated over a sliding window of size 100 kbp. Counts of the canonical telomeric sequences”TTAGGG”, and its reverse complement “CCCTAA” were found over a 50 kbp window. Spikes at the ends of scaffolds likely indicate the presence of telomeres. Scaffolds with telomere count spikes on either side can be found in *Paraconiothyrium Brasiliense strain* M42-189*, Nothophoma quercina* strain M97-236 *and Diplodia seriata strain* FDS-637, indicating that some of the scaffolds in these assemblies may be chromosomes sequence telomere-to-telomere. Functional annotations was assessed by evaluating the number of Gene Ontology (GO) terms (Fig. 2 and Tables S1–S3) and the Clusters of Orthologous Groups of proteins (COGs) present in each annotation, as shown in Fig. 3 and Table S4. Relatively similar percents of different COG groups and GO annotation terms were found between genomes of the same species.

### Phylogenomic analyses

**Fig. 4:**
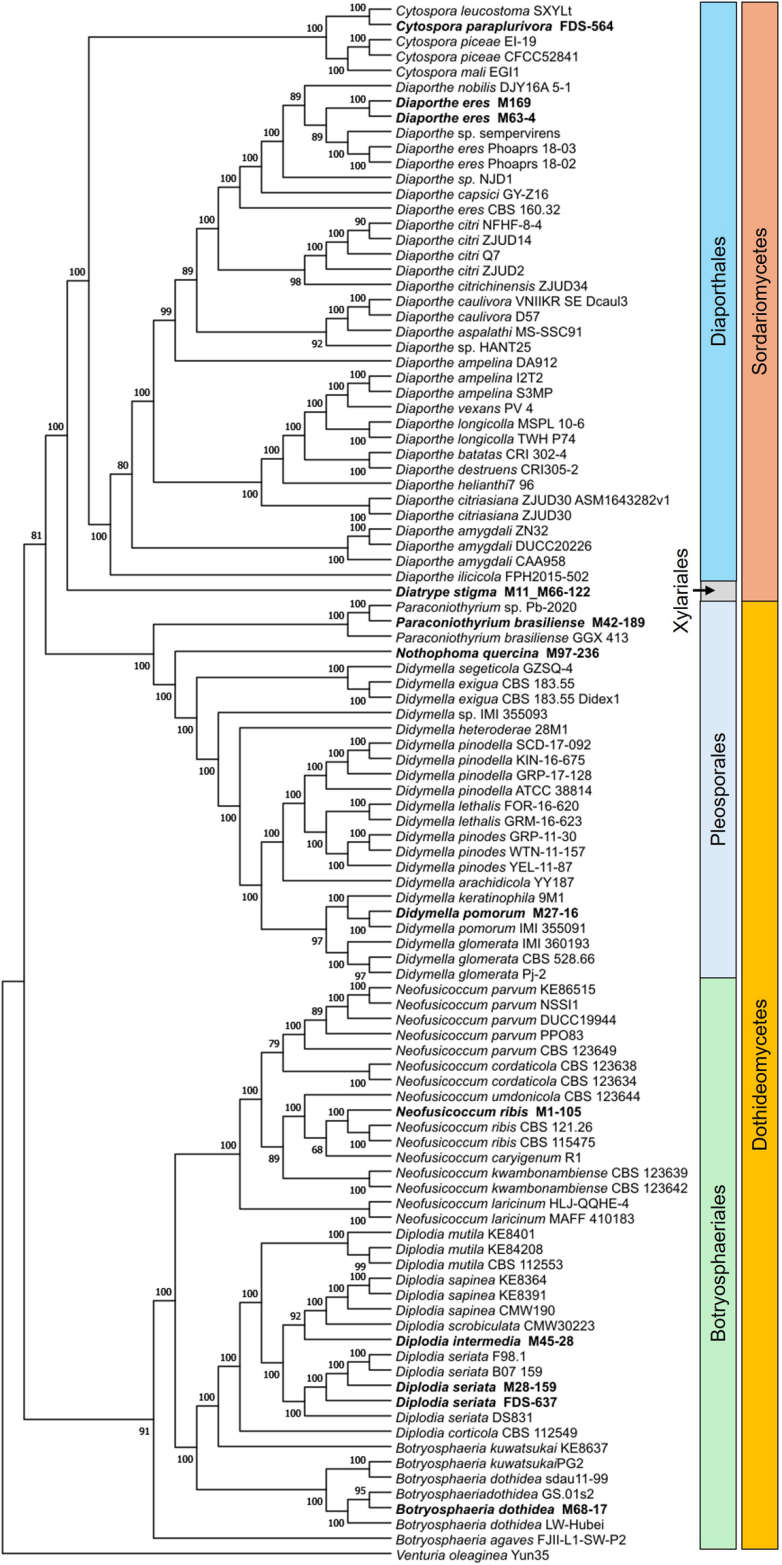
Maximum Likelihood phylogenetic tree generated using a concatenated alignment of 61 core genes extracted from the genomes of Dothideomycetes and Sordariomycetes species sourced from NCBI. The 12 fungal genomes obtained in this study are highlighted in bold. The tree was rooted to *Venturia oleaginea* Yun35. The numbers at each node represent bootstrap support, expressed as percentages.

Genomes of the 12 fungal strains sequenced, assembled, and annotated in this study, along with 90 fungal genomes downloaded from NCBI using the get-assemblies tool (GitHub - davised/get_assemblies), were included in the phylogenetic analysis (Fig. 4). All genomes were retrieved by using all genera names of the sequenced organisms in this study as queries, such as “*Didymella*”, “*Paraconiothyrium*”, and more. Sixty-one core genes, listed in Table 4, were extracted, aligned and concatenated using the Universal Fungal Core Genes (UFCG) pipeline version 1.0.5^54^. Maximum Likelihood-based phylogenetic analysis of the concatenated sequences was performed using IQ-Tree (v2.2.6)^55^, with 1,000 bootstrap replicates processed to determine the best-scoring Maximum Likelihood tree. The phylogenetic tree was rooted with *Venturia oleaginea* Yun35.

**Table 4:** List of the 61 core genes extracted from fungal genomes using the UFCG pipeline to generate the phylogenetic tree, along with their corresponding functions and identifiers sourced from the Saccharomyces Genome Database (SGD, www.yeastgenome.org) and UniProt (www.uniprot.org).

Gene	Function	SGD	UniProt
ACT1	Actin	YFL039C	P60010
ATP6	F1F0 ATP synthase subunit	Q0085	P00854
BMS1	Ribosome biogenesis protein	YPL217C	Q08965
BRE2	COMPASS component	YLR015W	P43132
CCT8	Chaperonin-containing T-complex subunit	YJL008C	P47079
CMD1	Calmodulin	YBR109C	P06787
COB	Cytochrome b	Q0105	P00163
COX1	Cytochrome c oxidase subunit	Q0045	P00401
COX2	Cytochrome c oxidase subunit	Q0250	P00410
COX3	Cytochrome c oxidase subunit	Q0275	P00420
DIP2	U3 small nucleolar RNA-associated protein 12	YLR129W	Q12220
DPH5	Diphthine methyl ester synthase	YLR172C	P32469
DYS1	Deoxyhypusine synthase	YHR068W	P38791
ELP3	Elongator complex protein 3	YPL086C	Q02908
ESF1	Pre-rRNA-processing protein	YDR365C	Q06344
FAP7	Adenylate kinase isoenzyme 6 homolog	YDL166C	Q12055
FRS1	Phenylalanine–tRNA ligase beta subunit	YLR060W	P15624
HEM12	Uroporphyrinogen decarboxylase	YDR047W	P32347
HIS4	Histidine biosynthesis trifunctional protein	YCL030C	P00815
HIS7	Imidazole glycerol phosphate synthase	YBR248C	P33734
ILV1	Threonine dehydratase	YER086W	P00927
KRE33	RNA cytidine acetyltransferase	YNL132W	P53914
MCM7	Mini-chromosome maintenance complex subunit	YBR202W	P38132
MET6	5-methyltetrahydropteroyltriglutamate–homocysteine methyltransferase	YER091C	P05694
MIP1	DNA polymerase gamma	YOR330C	P15801
MRPL19	54S ribosomal protein L19	YNL185C	P53875
MSF1	Phenylalanine–tRNA ligase	YPR047W	P08425
MSS51	Mitochondrial splicing suppressor protein 51	YLR203C	P32335
MVD1	Diphosphomevalonate decarboxylase	YNR043W	P32377
NCS6	Cytoplasmic tRNA 2-thiolation protein 1	YGL211W	P53088
NDI1	NADH-ubiquinone reductase	YML120C	P32340
NOG1	Nucleolar GTP-binding protein 1	YPL093W	Q02892
NOP14	Nucleolar complex protein 14	YDL148C	Q99207
OLI1	F0 ATP synthase subunit	Q0130	P61829
PAH1	Phosphatidate phosphatase	YMR165C	P32567
PGK1	Phosphoglycerate kinase	YCR012W	P00560
POL2	DNA polymerase epsilon catalytic subunit A	YNL262W	P21951
PRT1	Eukaryotic translation initiation factor 3 subunit B	YOR361C	P06103
RAD2	DNA repair protein	YGR258C	P07276
RLI1	Translation initiation factor	YDR091C	Q03195
RPB2	DNA-directed RNA polymerase II core subunit	YOR151C	P08518
RPF2	Ribosome biogenesis protein	YKR081C	P36160
RPN1	26S proteasome regulatory subunit	YHR027C	P38764
RPO21	DNA-directed RNA polymerase II core subunit	YDL140C	P04050
RPP0	60S acidic ribosomal protein P0	YLR340W	P05317
SDA1	Severe depolymerization of actin protein 1	YGR245C	P53313
SEC. 21	Coatomer subunit gamma	YNL287W	P32074
SEC. 26	Coatomer subunit beta	YDR238C	P41810
SPB1	27S pre-rRNA (guanosine(2922)-2’-O)-methyltransferase	YCL054W	P25582
TEF1	Translation elongation factor EF-1 alpha	YPR080W	P02994
TIF5	Eukaryotic translation initiation factor 5	YPR041W	P38431
TIM44	Mitochondrial import inner membrane translocase subunit	YIL022W	Q01852
TOP1	DNA topoisomerase 1	YOL006C	P04786
TRM1	tRNA (guanine(26)-N(2))-dimethyltransferase	YDR120C	P15565
TRP3	Multifunctional tryptophan biosynthesis protein	YKL211C	P00937
TSR1	Ribosome maturation factor	YDL060W	Q07381
TUB1	Alpha-tubulin	YML085C	P09733
TUB2	Beta-tubulin	YFL037W	P02557
UTP21	U3 small nucleolar RNA-associated protein 21	YLR409C	Q06078
VMA1	V-type proton ATPase catalytic subunit A	YDL185W	P17255
ZPR1	Zinc finger protein	YGR211W	P53303

## Data Records

The datasets have been deposited in the Sequenced Read Archive (SRA) under accession numbers: SRX14291869, SRX14155424, SRX14046716, SRX14003295, SRX13989866, SRX13979984, SRX13961038, SRX13940527, SRX13921100, SRX13894123, SRX13883208, SRX13877378, SRX13445800^56^. All assemblies and annotations can be found on NCBI GenBank under BioProject PRJNA790013^57^, with the following accession numbers: JAJVDB000000000^58^, JAJSPL000000000^59^, JAKOOP000000000^60^, JAKNSF000000000^61^, JAKJXP000000000^62^, JAKJXN000000000^63^, JAKEKT000000000^64^, JAJVDA000000000^65^, JAJVCZ000000000^66^, JAJVDC000000000^67^, JAKIXB000000000^68^, JAKJXO000000000^69^.

## Technical Validation

BUSCO (v5.8.2) employing the Ascomycota_odb12 dataset was used to assess whether core genes were adequately captured in the genomes sequenced. All genomes were of high quality (Table 5) and had greater than 97% of BUSCOs complete.

**Table 5:** Benchmarking Universal Copy Orthologs (BUSCO) assessment results.

	Complete BUSCOs	Complete single-copy BUSCOs	Complete duplicated BUSCOs	Fragmented BUSCOs	Missing BUSCOs	Percent Complete
*Botryosphaeria dothidea M68-17*	2771	2759	12	3	52	98.1%
*Cytospora paraplurivora FDS-564*	2780	2748	32	5	41	98.4%
*Diaporthe eres M169*	2778	2766	12	6	42	98.3%
*Diaporthe eres M63-4*	2776	2755	21	6	44	98.2%
*Diatrype stigma M11/M66-122*	2783	2776	7	5	38	98.5%
*Didymella pomorum M27-16*	2786	2779	7	5	35	98.6%
*Diplodia intermedia M45-28*	2771	2767	4	3	52	98.1%
*Diplodia seriata FDS-637*	2766	2761	5	5	55	97.9%
*Diplodia seriata M28-159*	2763	2760	3	4	59	97.8%
*Neofusicoccum ribis M1-105*	2769	2756	13	6	51	98.0%
*Nothophoma quercina M97-236*	2793	2788	5	5	28	98.8%
*Paraconiothyrium brasiliense M42-189*	2779	2771	8	13	34	98.3%

## Supplementary information

Supplementary Tables S1-S4

## Data Availability

Funannotate-based annotations were automated with the scripts available at the following gitub: https://github.com/Ellouzlab/Fun_pipeline. Bioinformatics programs were used as per their manual or protocol. If settings were omitted, default parameters were used.

## References

[1] Statistics Canada. *Area, production and farm gate value of marketed fruits: Table 32-10-0364-01*, 10.25318/3210036401-eng (2024).

[2] Ellouze, W. & Ilyukhin, E. First report of *Diplodia seriata* associated with canker and dieback diseases of apricot and nectarine trees in Ontario, Canada. *Journal of Plant Pathology* **106**, 759–760, 10.1007/s42161-023-01579-8 (2024).

[3] Ilyukhin, E. & Ellouze, W. First Report of *Neofusicoccum ribi*s causing cankers and dieback diseases on apricot trees in Canada and worldwide. *Plant Disease* **108**, 222, 10.1094/PDIS-08-23-1588-PDN (2024).

[4] Ellouze, W., Ilyukhin, E., Sulman, M. & Ali, S. First Report of *Diplodia intermedia* Causing Canker and Dieback Diseases on Apple Trees in Canada. *Plant Disease* **108**, 217, 10.1094/PDIS-07-23-1361-PDN (2024).

[5] Villanueva, O. & Ellouze, W. First report of a Canadian isolate of *Phytopythium vexans* causing root rot disease on apple and peach under laboratory conditions. *New Disease Reports* **48**, e12195, 10.1002/ndr2.12195 (2023).

[6] Ilyukhin, E., Schneider, K. & Ellouze, W. First Report of *Botryosphaeria dothidea* Causing Stem Canker and Dieback of Apple Trees in Ontario, Canada. *Plant Disease* **106**, 2994, 10.1094/PDIS-12-21-2838-PDN (2022).

[7] Ilyukhin, E. & Ellouze, W. First report of *Phaeobotryon negundinis* associated with twig and branch dieback of *Malus domestica* trees in southern Ontario, Canada and worldwide. *Journal of Plant Pathology* **105**, 355–356, 10.1007/s42161-022-01272-2 (2023).

[8] Ilyukhin, E., Nguyen, H. D. T., Castle, A. J. & Ellouze, W. *Cytospora paraplurivora* sp. nov. isolated from orchards with fruit tree decline syndrome in Ontario, Canada. *PLOS ONE* **18**, e0279490, 10.1371/journal.pone.0279490 (2023). 36630368 10.1371/journal.pone.0279490 PMC9833554

[9] Singh, J., Silva, K. J. P., Fuchs, M. & Khan, A. Potential role of weather, soil and plant microbial communities in rapid decline of apple trees. *PLOS ONE* **14**, e0213293, 10.1371/journal.pone.0213293 (2019). 30840713 10.1371/journal.pone.0213293 PMC6402675

[10] Stokstad, E. Rapid apple decline has researchers stumped. *Science* **363**, 1259–1259, 10.1126/science.363.6433.1259 (2019). 30898909 10.1126/science.363.6433.1259

[11] Ali, S., Renderos, W., Bevis, E., Hebb, J. & Abbasi, P. A. Diaporthe eres causes stem cankers and death of young apple rootstocks in Canada. *Canadian Journal of Plant Pathology* **42**, 218–227, 10.1080/07060661.2019.1653377 (2020).

[12] Wright, A. A., Szostek, S. A., Beaver-Kanuya, E. & Harper, S. J. Diversity of three bunya-like viruses infecting apple. *Archives of Virology* **163**, 3339–3343, 10.1007/s00705-018-3999-z (2018). 30132135 10.1007/s00705-018-3999-z

[13] Liu, H. *et al*. Characterization of a new apple luteovirus identified by high-throughput sequencing. *Virology journal* **15**, 85, 10.1186/s12985-018-0998-3 (2018). 29764461 10.1186/s12985-018-0998-3 PMC5952423

[14] Xiao, H., Hao, W., Storoschuk, G., MacDonald, J. L. & Sanfaçon, H. Characterizing the virome of apple orchards affected by rapid decline in the Okanagan and Similkameen valleys of British Columbia (Canada). *Pathogens* **11**, 1231, 10.3390/pathogens11111231 (2022). 36364981 10.3390/pathogens11111231 PMC9698585

[15] Gottwald, T. R., Wierenga, E., Luo, W. & Parnell, S. Epidemiology of Plum pox ‘D’ strain in Canada and the USA. *Canadian Journal of Plant Pathology* **35**, 442–457, 10.1080/07060661.2013.844733 (2013).

[16] Gougherty, A. V. & Nutter, F. W. Impact of eradication programs on the temporal and spatial dynamics of Plum pox virus on *Prunus* spp. in Pennsylvania and Ontario, Canada. *Plant Disease* **99**, 593–603, 10.1094/PDIS-03-14-0224-RE (2015). 30699685 10.1094/PDIS-03-14-0224-RE

[17] Parcey, M. *et al*. Comparative genomic analysis of *Erwinia amylovora* reveals novel insights in phylogenetic arrangement, plasmid diversity, and streptomycin resistance. *Genomics* **112**, 3762–3772, 10.1016/j.ygeno.2020.04.001 (2020). 32259573 10.1016/j.ygeno.2020.04.001

[18] Serrano, A. *et al*. The comparative root system architecture of declining and non-declining trees in two apple orchards in New York. *Plants* **12**, 2644, 10.3390/plants12142644 (2023). 37514258 10.3390/plants12142644 PMC10383163

[19] Xu, H., Hannam, K. D., MacDonald, J. L. & Ediger, D. Field investigation into tree fates from recent apple tree decline: Abrupt hydraulic failure versus gradual hydraulic loss. *Stresses* **3**, 256–269, 10.3390/stresses3010019 (2023).

[20] Termorshuizen, A. J. Ecology of Fungal Plant Pathogens. *Microbiol Spectr.* **4**, 1–11, 10.1128/microbiolspec.funk-0013-2016 (2016). 10.1128/microbiolspec.FUNK-0013-2016 28087933

[21] Aylward, J. *et al*. A plant pathology perspective of fungal genome sequencing. *IMA Fungus* **8**, 1–15, 10.5598/imafungus.2017.08.01.01 (2017). 28824836 10.5598/imafungus.2017.08.01.01 PMC5493528

[22] Kelman, A. Plant pathology at the crossroads. *Annual review of phytopathology* **23**, 1–12, 10.1146/annurev.py.23.090185.000245 (1985). 10.1146/annurev.py.23.090185.000245 22583073

[23] Hill, R. *et al*. Tapping culture collections for fungal endophytes: First genome assemblies for three genera and five species in the Ascomycota. *Genome Biology and Evolution* **15**, 10.1093/gbe/evad038 (2023). 10.1093/gbe/evad038 PMC10027605 36881851

[24] Villanueva, O., Nguyen, H. D. T. & Ellouze, W. Comparative genomic and secretome analysis of *Phytophthora capsici* strains: Exploring pathogenicity and evolutionary dynamics. *Agronomy* **14**, 2623 https://www.mdpi.com/2073-4395/14/11/2623 (2024).

[25] White, T. J., Bruns, T., Lee, S. & Taylor, J. in *PCR Protocols: A Guide to Methods and Applications* (eds Innis, M. A., Gelfand, D. H., Sninsky, J. J. & White, T. J.) 315–322 (Academic Press, 1990).

[26] Carbone, I. & Kohn, L. M. A method for designing primer sets for speciation studies in filamentous ascomycetes. *Mycologia* **91**, 553–556, 10.1080/00275514.1999.12061051 (1999).

[27] O’Donnell, K., Kistler, H. C., Cigelnik, E. & Ploetz, R. C. Multiple evolutionary origins of the fungus causing Panama disease of banana: Concordant evidence from nuclear and mitochondrial gene genealogies. *Proc. Natl. Acad. Sci.* **95**, 2044–2049, 10.1073/pnas.95.5.2044 (1998). 9482835 10.1073/pnas.95.5.2044 PMC19243

[28] Altschul, S. F., Gish, W., Miller, W., Myers, E. W. & Lipman, D. J. Basic local alignment search tool. *Journal of Molecular Biology* **215**, 403–410, 10.1016/S0022-2836(05)80360-2 (1990). 2231712 10.1016/S0022-2836(05)80360-2

[29] Koren, S. *et al*. Canu: scalable and accurate long-read assembly via adaptive k-mer weighting and repeat separation. *Genome Res* **27**, 722–736, 10.1101/gr.215087.116 (2017). 28298431 10.1101/gr.215087.116 PMC5411767

[30] Bankevich, A. *et al*. SPAdes: A new genome assembly algorithm and its applications to single-cell sequencing. *Journal of Computational Biology* **19**, 455–477, 10.1089/cmb.2012.0021 (2012). 22506599 10.1089/cmb.2012.0021 PMC3342519

[31] Li, H. & Durbin, R. Fast and accurate short read alignment with Burrows–Wheeler transform. *Bioinformatics* **25**, 1754–1760, 10.1093/bioinformatics/btp324 (2009). 19451168 10.1093/bioinformatics/btp324 PMC2705234

[32] Danecek, P. *et al*. Twelve years of SAMtools and BCFtools. *GigaScience* **10**, 10.1093/gigascience/giab008 (2021). 10.1093/gigascience/giab008 PMC7931819 33590861

[33] Walker, B. J. *et al*. Pilon: An integrated tool for comprehensive microbial variant detection and genome assembly improvement. *PLOS ONE* **9**, e112963, 10.1371/journal.pone.0112963 (2014). 25409509 10.1371/journal.pone.0112963 PMC4237348

[34] Mikheenko, A., Prjibelski, A., Saveliev, V., Antipov, D. & Gurevich, A. Versatile genome assembly evaluation with QUAST-LG. *Bioinformatics* **34**, i142–i150, 10.1093/bioinformatics/bty266 (2018). 29949969 10.1093/bioinformatics/bty266 PMC6022658

[35] Palmer, J. M. & Stajich, J. Funannotate v1.8.1: Eukaryotic genome annotation. *Zenodo, Zenodo.*10.5281/zenodo.4054262 (2020).

[36] Frith, M. C. A new repeat-masking method enables specific detection of homologous sequences. *Nucleic Acids Research* **39**, e23–e23, 10.1093/nar/gkq1212 (2010). 21109538 10.1093/nar/gkq1212 PMC3045581

[37] Waterhouse, R. M. *et al*. BUSCO Applications from Quality Assessments to Gene Prediction and Phylogenomics. *Mol Biol Evol* **35**, 543–548, 10.1093/molbev/msx319 (2018). 29220515 10.1093/molbev/msx319 PMC5850278

[38] Majoros, W. H., Pertea, M. & Salzberg, S. L. TigrScan and GlimmerHMM: two open source ab initio eukaryotic gene-finders. *Bioinformatics* **20**, 2878–2879, 10.1093/bioinformatics/bth315 (2004). 15145805 10.1093/bioinformatics/bth315

[39] Korf, I. Gene finding in novel genomes. *BMC Bioinformatics* **5**, 59, 10.1186/1471-2105-5-59 (2004). 15144565 10.1186/1471-2105-5-59 PMC421630

[40] Stanke, M., Diekhans, M., Baertsch, R. & Haussler, D. Using native and syntenically mapped cDNA alignments to improve *de novo* gene finding. *Bioinformatics* **24**, 637–644, 10.1093/bioinformatics/btn013 (2008). 18218656 10.1093/bioinformatics/btn013

[41] Borodovsky, M. & Lomsadze, A. Eukaryotic gene prediction using GeneMark.hmm-E and GeneMark-ES. *Current Protocols in Bioinformatics* **35**, 4.6.1–4.6.10, 10.1002/0471250953.bi0406s35 (2011). 10.1002/0471250953.bi0406s35 PMC3204378 21901742

[42] Haas, B. J. *et al*. Automated eukaryotic gene structure annotation using EVidenceModeler and the Program to Assemble Spliced Alignments. *Genome Biology* **9**, R7, 10.1186/gb-2008-9-1-r7 (2008). 18190707 10.1186/gb-2008-9-1-r7 PMC2395244

[43] Chan, P. P. & Lowe, T. M. in *Gene Prediction: Methods and Protocols* (ed Kollmar, M.) 1–14 (Springer New York, 2019).

[44] Blin, K. *et al*. antiSMASH 6.0: improving cluster detection and comparison capabilities. *Nucleic Acids Research* **49**, W29–W35, 10.1093/nar/gkab335 (2021). 33978755 10.1093/nar/gkab335 PMC8262755

[45] Quevillon, E. *et al*. InterProScan: protein domains identifier. *Nucleic Acids Res* **33**, W116–120, 10.1093/nar/gki442 (2005). 15980438 10.1093/nar/gki442 PMC1160203

[46] Cantalapiedra, C. P., Hernández-Plaza, A., Letunic, I., Bork, P. & Huerta-Cepas, J. eggNOG-mapper v2: Functional annotation, orthology assignments, and domain prediction at the metagenomic scale. *Molecular Biology and Evolution* **38**, 5825–5829, 10.1093/molbev/msab293 (2021). 34597405 10.1093/molbev/msab293 PMC8662613

[47] Huerta-Cepas, J. *et al*. eggNOG 5.0: a hierarchical, functionally and phylogenetically annotated orthology resource based on 5090 organisms and 2502 viruses. *Nucleic Acids Res* **47**, D309–d314, 10.1093/nar/gky1085 (2019). 30418610 10.1093/nar/gky1085 PMC6324079

[48] El-Gebali, S. *et al*. The Pfam protein families database in 2019. *Nucleic Acids Research* **47**, D427–D432, 10.1093/nar/gky995 (2019). 30357350 10.1093/nar/gky995 PMC6324024

[49] Yin, Y. *et al*. dbCAN: a web resource for automated carbohydrate-active enzyme annotation. *Nucleic Acids Res* **40**, W445–451, 10.1093/nar/gks479 (2012). 22645317 10.1093/nar/gks479 PMC3394287

[50] Rawlings, N. D., Tolle, D. P. & Barrett, A. J. MEROPS: the peptidase database. *Nucleic Acids Res* **32**, D160–164, 10.1093/nar/gkh071 (2004). 14681384 10.1093/nar/gkh071 PMC308805

[51] Cantarel, B. L. *et al*. The Carbohydrate-Active EnZymes database (CAZy): an expert resource for Glycogenomics. *Nucleic Acids Research* **37**, D233–D238, 10.1093/nar/gkn663 (2009). 18838391 10.1093/nar/gkn663 PMC2686590

[52] Käll, L., Krogh, A. & Sonnhammer, E. L. A combined transmembrane topology and signal peptide prediction method. *J Mol Biol* **338**, 1027–1036, 10.1016/j.jmb.2004.03.016 (2004). 15111065 10.1016/j.jmb.2004.03.016

[53] Teufel, F. *et al*. SignalP 6.0 predicts all five types of signal peptides using protein language models. *Nat Biotechnol* **40**, 1023–1025, 10.1038/s41587-021-01156-3 (2022). 34980915 10.1038/s41587-021-01156-3 PMC9287161

[54] Kim, D., Gilchrist, C. L. M., Chun, J. & Steinegger, M. UFCG: database of universal fungal core genes and pipeline for genome-wide phylogenetic analysis of fungi. *Nucleic Acids Research* **51**, D777–D784, 10.1093/nar/gkac894 (2022). 10.1093/nar/gkac894 PMC9825530 36271795

[55] Minh, B. Q. *et al*. IQ-TREE 2: New models and efficient methods for phylogenetic inference in the genomic era. *Molecular Biology and Evolution* **37**, 1530–1534, 10.1093/molbev/msaa015 (2020). 32011700 10.1093/molbev/msaa015 PMC7182206

[56] Sulman, M. *et al*. Fungi associated with Fruit Tree Decline symptoms in Ontario, Canada. *NCBI Sequenced Read Archive* https://identifiers.org/ncbi/insdc.sra:SRP351463 (2024).

[57] Sulman, M. *et al*. Fungi associated with Fruit Tree Decline symptoms in Ontario, Canada. *NCBI BioProject* https://www.ncbi.nlm.nih.gov/bioproject/PRJNA790013/ (2024).

[58] Sulman, M., Ilyukhin, E., Ali, S. & Ellouze, W. *Botryosphaeria dothidea* strain M68-17, whole genome shotgun sequencing project. *GenBank* https://identifiers.org/ncbi/insdc:JAJVDB000000000 (2024).

[59] Sulman, M., Ilyukhin, E., Nguyen, H. D. T., Ali, S. & Ellouze, W. *Cytospora paraplurivora* strain FDS-564, whole genome shotgun sequencing project. *GenBank* https://identifiers.org/ncbi/insdc:JAJSPL000000000 (2024).

[60] Sulman, M., Ilyukhin, E., Nguyen, H. D. T., Ali, S. & Ellouze, W. *Diaporthe eres* strain M63-4, whole genome shotgun sequencing project. *GenBank* https://identifiers.org/ncbi/insdc:JAKOOP000000000 (2024).

[61] Sulman, M., Ilyukhin, E., Ali, S. & Ellouze, W. *Diaporthe eres* strain M169, whole genome shotgun sequencing project. *GenBank* https://identifiers.org/ncbi/insdc:JAKNSF000000000 (2024).

[62] Sulman, M., Ilyukhin, E., Ali, S. & Ellouze, W. *Diatrype stigma* strain M11/M66-122, whole genome shotgun sequencing project. *GenBank* https://identifiers.org/ncbi/insdc:JAKJXP000000000 (2024).

[63] Sulman, M., Ilyukhin, E., Ali, S. & Ellouze, W. *Didymella pomorum* strain M27-16, whole genome shotgun sequencing project. *GenBank* https://identifiers.org/ncbi/insdc:JAKJXN000000000 (2024).

[64] Sulman, M., Ilyukhin, E., Ali, S. & Ellouze, W. *Diplodia intermedia* strain M45-28, whole genome shotgun sequencing project. *GenBank* https://identifiers.org/ncbi/insdc:JAKEKT000000000 (2024).

[65] Sulman, M., Ilyukhin, E., Ali, S. & Ellouze, W. *Diplodia seriata* strain M28-159, whole genome shotgun sequencing project. *GenBank* https://identifiers.org/ncbi/insdc:JAJVDA000000000 (2024).

[66] Sulman, M., Ilyukhin, E., Nguyen, H. D. T., Ali, S. & Ellouze, W. *Diplodia seriata* strain FDS-637, whole genome shotgun sequencing project. https://identifiers.org/ncbi/insdc:JAJVCZ000000000 (2024).

[67] Sulman, M., Ilyukhin, E., Ali, S. & Ellouze, W. *Neofusicoccum ribis* strain M1-105, whole genome shotgun sequencing project. *GenBank* https://identifiers.org/ncbi/insdc:JAJVDC000000000 (2024).

[68] Sulman, M., Ilyukhin, E., Ali, S. & Ellouze, W. *Nothophoma quercina* strain M97-236, whole genome shotgun sequencing project. *GenBank* https://identifiers.org/ncbi/insdc:JAKIXB000000000 (2024).

[69] Sulman, M., Ilyukhin, E., Ali, S. & Ellouze, W. *Paraconiothyrium brasiliense* strain M42-189, whole genome shotgun sequencing project. *GenBank* https://identifiers.org/ncbi/insdc:JAKJXO000000000 (2024).

