# Supplementary material for: De novo Genome Assembly and Annotation of 12 Fungi Associated with Fruit Tree Decline Syndrome in ON, Canada
Source: Sci Data. 2025 Jul 1;12:1098. doi: 10.1038/s41597-025-05192-5 (PMC12217926; doi:10.1038/s41597-025-05192-5)
Supplement: Supplementary file 1 — Supplementary Tables S1-S4 [file 41597_2025_5192_MOESM1_ESM.pdf]

Table S1. Counts of genes annotated with the top 15 most frequent Gene Ontology (GO) molecular function terms, plus an "other" category.

Assembly	Oxidation-reduction process	Trans-membrane transport	Metabolic process	Carbohydrate metabolic process	Regulation of transcription, DNA-templated	Transcription, DNA-templated	Proteolysis	Protein phos-phorylation	Transport	Translation	DNA repair	Signal transduction	Intracellular protein transport	Lipid metabolic process	Biosynthetic process	Other
Botryosphaeria dothidea strain M68-17	1203	617	498	279	218	169	151	131	155	108	66	51	54	51	56	2589
Cytospora paraplurivora strain FDS-564	733	427	339	220	151	109	120	117	109	111	67	52	54	44	46	2402
Diaporthe eres strain M169	1435	713	556	361	178	170	201	172	150	109	70	56	52	62	66	2719
Diaporthe eres strain M63-4	1432	693	564	367	172	153	198	158	152	110	69	58	51	61	63	2700
Diatrype stigma strain M11/M66-122	961	491	373	258	169	130	147	132	117	105	66	48	52	39	48	2442
Didymella pomorum strain M27-16	805	525	360	267	132	118	121	113	117	112	72	61	55	52	36	2502
Diplodia intermedia strain M45-28	849	492	385	252	213	146	113	133	126	115	67	52	53	43	43	2498
Diplodia seriata strain FDS-637	844	488	360	243	182	134	112	131	128	106	60	52	53	43	42	2430
Diplodia seriata strain M28-159	825	487	361	246	180	141	122	127	127	108	60	54	55	43	42	2408
Neofusicoccum ribis strain M1-105	1171	636	486	282	210	176	147	146	155	111	68	51	53	58	48	2587
Nothophoma quercina strain M97-236	756	503	352	249	123	113	121	113	118	108	68	55	56	55	37	2433
Paraconiothyrium brasiliense strain M42-189	989	543	401	311	142	144	146	137	133	100	69	54	54	50	51	2478

Table S2. Counts of genes annotated with the top 15 most frequent GO cellular component terms, plus an "other" category.

Assembly	Protein binding	Oxidoreductase activity	Catalytic activity	ATP binding	Zinc ion binding	DNA binding	Nucleic acid binding	heme binding	iron ion binding	oxidoreductase activity, acting on paired donors, with incorporation or reduction of molecular oxygen	hydrolase activity	hydrolase activity, hydrolyzing O-glycosyl compounds	metal ion binding	flavin adenine dinucleotide binding	nucleotide binding	Other
Botryosphaeria dothidea strain M68-17	784	848	758	522	422	305	292	303	297	271	223	169	171	167	150	5424
Cytospora paraplurivora strain FDS-564	643	542	534	469	283	252	259	148	144	132	149	108	123	113	138	4414
Diaporthe eres strain M169	828	1018	853	589	419	318	276	384	377	348	257	212	164	242	158	5935
Diaporthe eres strain M63-4	803	998	849	588	397	302	271	392	388	357	263	213	150	229	162	5916
Diatrype stigma strain M11/M66-122	746	714	638	511	353	265	266	218	210	196	177	150	154	170	150	4777
Didymella pomorum strain M27-16	670	631	575	462	309	247	254	152	136	121	165	155	146	135	152	4701
Diplodia intermedia strain M45-28	776	629	594	485	377	278	290	168	164	141	181	152	163	113	146	4803
Diplodia seriata strain FDS-637	734	610	560	487	344	254	276	174	165	143	169	148	146	117	140	4616
Diplodia seriata strain M28-159	747	597	562	488	339	264	284	169	164	142	168	151	152	108	141	4621
Neofusicoccum ribis strain M1-105	760	831	730	528	422	315	290	305	305	276	220	170	174	163	149	5375
Nothophoma quercina strain M97-236	677	599	561	450	306	237	240	133	118	100	160	144	142	119	144	4569
Paraconiothyrium brasiliense strain M42-189	806	732	674	504	371	275	258	226	222	197	194	182	157	153	142	4995

Table S3. Counts of genes annotated with the top 15 most frequent GO biological process terms, plus an "other" category.

Assembly	Integral component of membrane	Membrane	Nucleus	Cytoplasm	intracellular	ribosome	extracellular region	mitochondrion	mediator complex	endoplasmic reticulum	proteasome core complex	eukaryotic translation initiation factor 3 complex	endoplasmic reticulum membrane	mitochondrial inner membrane	cell wall	Other
Botryosphaeria dothidea strain M68-17	716	385	355	134	110	97	27	21	21	15	14	13	12	11	11	568
Cytospora paraplurivora strain FDS-564	507	261	257	126	117	100	14	21	17	17	14	13	13	11	11	543
Diaporthe eres strain M169	801	387	335	140	115	96	38	20	18	14	14	13	14	12	19	565
Diaporthe eres strain M63-4	779	392	317	140	112	95	35	20	19	15	14	13	14	13	18	563
Diatrype stigma strain M11/M66-122	587	302	294	123	108	95	26	21	19	17	14	12	14	11	9	546
Didymella pomorum strain M27-16	638	342	260	135	113	100	36	22	15	15	14	13	12	10	6	537
Diplodia intermedia strain M45-28	599	328	323	133	112	102	25	20	19	14	14	13	12	12	10	559
Diplodia seriata strain FDS-637	585	321	304	124	106	94	21	21	19	13	13	13	12	11	10	532
Diplodia seriata strain M28-159	585	316	295	127	105	96	22	21	19	14	14	13	12	12	9	536
Neofusicoccum ribis strain M1-105	738	391	355	141	112	99	32	22	19	14	14	13	13	11	15	565
Nothophoma quercina strain M97-236	605	322	249	126	109	96	36	18	18	15	14	13	8	11	6	536
Paraconiothyrium brasiliense strain M42-189	632	341	292	128	107	87	28	17	19	13	14	13	9	13	10	529

Table S4. Counts of genes annotated with all detected Clusters of Orthologous Groups (COGs) categories across fungal genomes. Categories include: (A) RNA processing and modification; (B) Chromatin structure and dynamics; (C) Energy production and conversion; (D) Cell cycle control and mitosis; (E) Amino acid transport and metabolism; (F) Nucleotide transport and metabolism; (G) Carbohydrate transport and metabolism; (H) Coenzyme transport and metabolism; (I) Lipid transport and metabolism; (J) Translation, ribosomal structure and biogenesis; (K) Transcription; (L) Replication and repair; (M) Cell wall/membrane biogenesis; (N) Cell motility; (O) Post-translational modification, protein turnover, chaperones; (P) Inorganic ion transport and metabolism; (Q) Secondary metabolites biosynthesis, transport, and catabolism; (S) Function unknown; (T) Signal transduction mechanisms; (U) Intracellular trafficking and secretion; (V) Defense mechanisms; (W) Extracellular structures; (Y) Nuclear structure; (Z) Cytoskeleton.

	A	B	C	D	E	F	G	H	I	J	K	L	M	N	O	P	Q	S	T	U	V	W	Y	Z
Botryosphaeria dothidea strain M68-17	**302**	**147**	**491**	**162**	**675**	**134**	**811**	**234**	**433**	**403**	**386**	**254**	**124**	**6**	**651**	**329**	**844**	**2827**	**387**	**482**	**62**	**6**	**27**	**126**
Cytospora paraplurivora strain FDS-564	**305**	**119**	**366**	**166**	**463**	**99**	**584**	**232**	**343**	**384**	**333**	**243**	**77**	**5**	**555**	**205**	**509**	**2197**	**364**	**447**	**43**	**5**	**28**	**125**
Diaporthe eres strain M169	**321**	**168**	**556**	**179**	**743**	**144**	**1040**	**315**	**473**	**420**	**386**	**285**	**143**	**5**	**779**	**329**	**1004**	**3300**	**435**	**508**	**82**	**7**	**27**	**133**
Diaporthe eres strain M63-4	**318**	**176**	**567**	**188**	**741**	**145**	**1056**	**320**	**484**	**428**	**395**	**287**	**138**	**6**	**795**	**335**	**1040**	**3296**	**446**	**518**	**80**	**6**	**27**	**133**
Diatrype stigma strain M11/M66-122	**302**	**143**	**455**	**172**	**563**	**116**	**727**	**245**	**360**	**412**	**383**	**257**	**98**	**5**	**717**	**240**	**660**	**2612**	**361**	**481**	**54**	**6**	**27**	**131**
Didymella pomorum strain M27-16	**301**	**122**	**375**	**158**	**522**	**110**	**754**	**193**	**337**	**384**	**345**	**247**	**90**	**6**	**591**	**310**	**507**	**2318**	**372**	**449**	**41**	**6**	**24**	**128**
Diplodia intermedia strain M45-28	**314**	**141**	**394**	**166**	**571**	**118**	**700**	**204**	**345**	**395**	**377**	**247**	**112**	**6**	**604**	**287**	**560**	**2578**	**401**	**465**	**57**	**7**	**28**	**124**
Diplodia seriata strain FDS-637	**296**	**134**	**394**	**165**	**564**	**116**	**691**	**200**	**353**	**388**	**352**	**245**	**102**	**6**	**594**	**289**	**553**	**2386**	**382**	**449**	**56**	**7**	**29**	**117**
Diplodia seriata strain M28-159	**303**	**137**	**398**	**167**	**554**	**116**	**697**	**200**	**352**	**388**	**366**	**247**	**101**	**8**	**590**	**286**	**548**	**2419**	**383**	**462**	**59**	**7**	**29**	**117**
Neofusicoccum ribis strain M1-105	**307**	**142**	**484**	**163**	**670**	**130**	**819**	**240**	**457**	**406**	**386**	**258**	**121**	**6**	**646**	**330**	**818**	**2784**	**398**	**482**	**75**	**6**	**30**	**125**
Nothophoma quercina strain M97-236	**300**	**126**	**357**	**169**	**518**	**106**	**717**	**192**	**341**	**383**	**336**	**244**	**93**	**4**	**605**	**307**	**480**	**2294**	**380**	**461**	**45**	**7**	**25**	**130**
Paraconiothyrium brasiliense strain M42-189	**304**	**150**	**420**	**169**	**573**	**116**	**826**	**214**	**420**	**397**	**374**	**252**	**124**	**5**	**666**	**307**	**708**	**2638**	**399**	**502**	**65**	**10**	**30**	**135**
